# Cross-sectional association between brain-derived neurotrophic factor and intrinsic capacity in older adults: The mediating role of oxidative stress

**DOI:** 10.1016/j.jnha.2025.100599

**Published:** 2025-06-12

**Authors:** Chi Zhang, Ping Zeng, Yushan Zhang, Yuting Kang, Jie Zhang, Jing Li, Hong Shi, Shiwei Liu, Ji Shen

**Affiliations:** a Tobacco Control Office, Chinese Center for Disease Control and Prevention, Beijing, China; b The Key Laboratory of Geriatrics, Beijing Institute of Geriatrics, Beijing Hospital, National Center of Gerontology, National Health Commission, Institute of Geriatric Medicine, Chinese Academy of Medical Sciences, Department of Geriatrics, Beijing, China; c Department of Geriatrics, Beijing Hospital, National Center of Gerontology, Institute of Geriatric Medicine, Chinese Academy of Medical Sciences, Beijing, China; d Department of Scientific Research, Beijing Hospital, National Center of Gerontology, Institute of Geriatric Medicine, Chinese Academy of Medical Sciences, Beijing, China

**Keywords:** Older adults, Intrinsic capacity, Brain-derived neurotrophic factor, Oxidative stress

## Abstract

**Objectives:**

Brain-derived neurotrophic factor (BDNF) is a key indicator in the brain-muscle axis. This study aimed to investigate the association of plasma BDNF and intrinsic capacity (IC) in older people, and to examine the mediating role of inflammation and oxidative stress.

**Methods:**

This cross-sectional study included 658 community-dwelling older adults (70.38 ± 6.06 years, 59.42% female). Intrinsic capacity including five domains was evaluated according to the World Health Organization recommendation. Plasma BDNF, high sensitivity C-reactive protein (hs-CRP), Interleukin-6 (IL-6), Tumor necrosis factor-alpha (TNF-a), Interleukin-1 beta (IL-1β), Malondialdehyde (MDA), Superoxide dismutase (SOD), Glutathione peroxidase (GSH-Px), and Glutathione reductase (GR)were measured by enzyme-linked immunosorbent assay methods. Restricted cubic spline and multivariate logistic regression were conducted to explore the association of BDNF with IC impairment. Mediation analyses were used to explore the potential mechanisms. Demographic characteristics, health behaviors, and comorbidities were included as covariates.

**Results:**

247(37.54%) participants had IC impairment. Older individuals with impaired IC had lower levels of BDNF, IL-1β, SOD, and GR, while showed higher levels of hs-CRP and MDA compared to the normal group. There was an L-shaped negative correlation between BDNF levels and the odds of IC impairment (*P*-nonlinear <0.001). After adjusting for all confounders, the odds for IC impairment in the medium and high BDNF tertiles were significantly lower than in the low BDNF tertile, with ORs of 0.43(95% CI: 0.26−0.89, *P* = 0.004) and 0.38(95% CI: 0.20−0.71, *P* = 0.007), respectively. Plasma SOD and GR mediated 4.13% (95% CI: 1.15, 7.16) and 7.82% (95% CI: 3.24, 12.48) of the total effect of BDNF on IC.

**Conclusion:**

High levels of circulatory BDNF may be related to lower odds of IC impairment. Oxidative stress status partially explains the mechanisms underlying the association.

## 1 Introduction

Intrinsic capacity (IC) is an important concept proposed by the World Health Organization (WHO) in 2015, which refers to an individual's ability to integrate multiple aspects, including locomotion, psychology, cognition, sensory function and vitality [1]. Reduced levels of IC are associated with an elevated risk of disability [2], institutionalization [3], and mortality [4,5]. Therefore, examining factors associated with IC, including health lifestyles and biological markers, is important for understanding the potential mechanism and informing strategies to prevent functional decline among older adults.

In recent years, cross-sectional and longitudinal studies have focused on biological markers related to IC and its dimensions [6,7]. Lu et al. found that elevated plasma inflammation indicators such as C-reactive protein (CRP), interleukin-6 (IL-6), and growth differentiation factor-15 were negatively associated with IC at baseline [8,9]. Previous studies have also focused on the effect of oxidative stress and metabolic markers such as insulin-like growth factor [10], leptin [11], and superoxide dismutase (SOD) [12]. Considering that cognitive ability and skeletal muscle function are core dimensions of IC, novel indicators related to the neuromuscular junction still need to be identified to elucidate the underlying mechanisms of IC changes. Brain-derived neurotrophic factor (BDNF) is a neurotrophic myokine that is widely distributed in the central nervous system and peripheral tissues [13,14]. BDNF is not only crucial for brain health but is also closely associated with skeletal muscle growth, energy metabolism, and cardiovascular function, serving as an essential molecular basis for maintaining overall functional health [15,16].

Previous studies have confirmed the cross-sectional associations of circulating BDNF levels with cognitive reserve [17] and age‑related memory impairment [18] in older adults. Other studies have shown that circulating BDNF is associated with frailty and sarcopenia, yet the results are inconsistent [19,20]. To our knowledge, even though BDNF is beneficial to the function of multiple systems in the body, there are very few studies reporting the effects and mechanisms of BDNF on IC in community-dwelling older adults. BDNF exerts its antioxidant effects by activating AMPK and PGC-1α, thereby enhancing fatty acid oxidation and reducing oxidative stress [15]. Besides, proinflammatory cytokines and BDNF often exhibit inverse changes during the process of neurodegeneration. For instance, Noren et al. found that BDNF was negatively associated with hs-CRP in women, and may have a protective role in counteracting inflammation [21]. These findings suggest that both decreased BDNF levels and oxidative stress may be implicated in the pathophysiology of neurodegenerative diseases. However, whether inflammation and oxidative stress mediate the association between BDNF and functional decline in older individuals remains unclear.

This study aims to provide novel biological insights into the associated factors of IC impairment. We hypothesize that plasma BDNF levels may be positively correlated with IC in older adults. Additionally, we will also investigate whether inflammation and oxidative stress mediate this relationship.

## 2 Methods

### 2.1 Study population

Between February and August 2023, a total of 937 participants aged ≥60 years were initially recruited from four communities in Beijing. All procedures, including questionnaire surveys, physical examinations, and biological sample collections, were conducted by nurses who had undergone unified training. The inclusion criteria were: (1) age ≥ 60 years; (2) clear consciousness and no communication barriers with the interviewer; (3) voluntary participation in the study and cooperation in completing the questionnaire and physical examination. After sequentially excluding participants who did not provide blood samples (n = 233), those with missing IC data (n = 9), those with undeterminable BDNF data due to low abundance (n = 8), and those with missing covariates (n = 29), a total of 658 participants were included in the final analysis. There were no statistically significant differences in age, gender, ethnicity, educational level, marital status, smoking status, and alcohol consumption between the included samples and the excluded samples (Supplement Table S1).

### 2.2 Assessment of IC and dimensions

In accordance with the WHO guidelines, five dimensions were measured and combined to establish the overall IC score: (1) locomotion was evaluated using the Short Physical Performance Battery (SPPB); (2) cognition was assessed by the Mini-Mental State Examination (MMSE); (3) vitality was assessed by the Mini-Nutritional Assessment Short Form (MNA-SF); (4) psychology dimension was assessed by the Patient Health Questionnaire-9 (PHQ-9); (5) Vision was measured using the international visual acuity chart, with normal vision defined as uncorrected visual acuity ≥0.8 in both eyes; hearing was assessed using the whispered voice test, with normal hearing defined as passing the test in both ears. The classification of each dimension and its corresponding IC score are shown in Table 1. The global IC score ranged from 0 to 10 points, and participants were divided into the normal IC group (9–10 points) and impaired IC group (0–8 points) with reference to previous studies [5,22].

**Table 1:** Measurement and classification of five IC dimensions.

Dimension	Grades	IC score
Locomotion	SPBB: 10–12 points	2
	SPBB: 3–9 points	1
	SPBB: 0–2 points	0
Cognition	MMSE: 27–30 points	2
	MMSE: 10–26 points	1
	MMSE: 0–9 points	0
Vitality	MNA-SF: 12−14 points	2
	MNA-SF: 8−11 points	1
	MNA-SF: 0−7 points	0
Psychological	PHQ-9: 0–4 points	2
	PHQ-9: 5–9 points	1
	PHQ-9: 10–27 points	0
Sensory	Vision and hearing normal	2
	Vision or hearing impaired	1
	Vision and hearing impaired	0

### 2.3 Blood samples

Venous blood samples (5 ml) were collected from each participant before breakfast. The samples were immediately processed by centrifugation at 20 °C and 500 rpm for 10 min to separate the plasma. The plasma was then transported to the Beijing Hospital Biological Sample Bank via a cold chain and stored at −80 °C for the subsequent detection of BDNF, inflammatory factors and oxidative stress indicators.

### 2.4 Measurement of BDNF

Plasma BDNF levels were determined using an enzyme-linked immunosorbent assay (ELISA) with a commercial kit (RayBiotech, USA). The inter-assay and intra-assay coefficients of variation (CV) were 2.7% and 4.2%. Due to the lack of normal reference value ranges for BDNF, participants were categorized into three groups based on BDNF concentration tertiles: high level (≥7.28 µg/L), medium level (≥1.34 µg/L and <7.28 µg/L), and low level (<1.34 µg/L).

### 2.5 Covariates

The following covariates were considered based on previous studies [2,[7], [8], [9],^20^,[23], [24], [25]]. Data on age (as a continuous variable), sex (female or male), ethnicity (Han ethnicity or others), education (primary school or below, junior or senior school, or undergraduate or above), living arrangement (married and living together or not), household income (<10,000 yuan, ≥10,000 and <30,000 yuan, or ≥30,000 yuan), current smoking (yes or no), and current alcohol consumption (yes or no) were collected through standardized questionnaires during face-to-face interviews. Regular physical exercise was assessed based on the dimension of reduced physical activity in the Fried frailty phenotype [men: <383 kcal/week (approximately equivalent to walking for 2.5 h); women: <270 kcal/week (approximately equivalent to walking for 2 h)]. Body mass index (BMI) was calculated as weight in kilograms divided by the square of height in meters (kg/m²). Activities of daily living (ADL) were evaluated using the Barthel Index, with a total score of <95 indicating ADL impairment. Charlson comorbidity index was used to assess the presence of 28 common chronic diseases.

### 2.6 Candidate mediators

A series of circulating inflammatory and oxidative stress markers were measured using ELISA with commercial kits (RayBiotech, USA) were considered as candidate mediators, including hs-CRP, IL-6, TNF-α, IL-1β, malondialdehyde (MDA), superoxide dismutase (SOD), glutathione reductase (GR), and glutathione peroxidase (GSH-Px). The inter-assay and intra-assay CVs of these mediators ranged were 1.9–7.1 % and 2.6–8.6%, respectively.

### 2.7 Statistical analyses

Normally distributed continuous data were described as mean ± standard deviation (Mean ± SD), while non-normally distributed continuous data were presented as median and interquartile range [Median (P_25_, P_75_)]. Categorical data were described using frequency (percentage) [n (%)]. Differences in sample characteristics among participants with different IC scores were compared using Student’s t-test, Wilcoxon rank-sum test, or chi-square test. Spearman correlation coefficient was used to examine the associations between BDNF levels and intrinsic capacity and its dimensions. The dose-response relationship between BDNF levels and the risk of IC impairment was analyzed using restricted cubic spline (RCS) regression. The relationship between BDNF and IC impairment was assessed using multivariable binary logistic regression, adjusting for demographic characteristics, lifestyle factors, and chronic diseases. In each logistic regression model, odds ratios (OR) and 95% confidence intervals (CI) were calculated. The predictive ability of BDNF for IC impairment was evaluated using receiver operating characteristic (ROC) curve analysis, and an area under the curve (AUC) ≥ 0.7 was considered good predictive ability. Structural equation modeling (SEM) with partial least squares estimation was used to test the mediating roles of inflammation and oxidative stress in the relationship between BDNF and IC. A bias-corrected bootstrap method was employed to obtain the significance of the indirect effect, with 500 random samples resampled from the original dataset to calculate the 95% CIs. Mediation effects were considered statistically significant if the 95% CI of proportion mediated (PM) did not include 0. All statistical analyses were performed using R version 4.2.0. A two-sided p-value of less than 0.05 was considered statistically significant.

## 3 Results

### 3.1 Sample characteristics

The mean age of 658 older adults was 70.38 ± 6.06 years, and 59.42% were female. 247 (47.54%) participants had impaired IC. The mean BDNF level of the sample was 3.33 (0.73, 9.98) µg/L, which was significantly higher in the normal IC group than in the impaired IC group [5.28 (1.59, 13.37) µg/L vs. 1.22 (0.37, 4.21) µg/L, Z = −9.371, *P* < 0.001]. Compared to the normal group, participants in the impaired IC group exhibited higher age, lower family income, lower education level, reduced physical activities, and more severe comorbidity conditions (*P*s < 0.05). Older individuals with impaired IC had lower levels of BDNF, IL-1β, SOD and GR, but showed higher levels of hs-CRP and MDA compared to the normal group. Sample characteristics of all participants across IC scores are shown in Table 2.

**Table 2:** Characteristics and BDNF levels of 658 older adults.

Sample characteristics	Total sample (n = 658)	IC normal (n = 411)	IC impaired (n = 247)	t/Z/χ^2^	*P* value
Age (M ± SD, years)	70.38 ± 6.06	68.94 ± 5.59	72.81 ± 6.06	8.311	<0.001
Female [n (%)]	391 (59.42)	234 (56.93)	157 (63.56)	2.826	0.092
Han ethnicity [n (%)]	617 (93.77)	390 (94.89)	227 (91.90)	2.292	0.131
Education level [n (%)]				42.713	<0.001
primary school or below	24 (3.65)	4 (0.97)	20 (8.10)		
junior or senior school	433 (65.81)	253 (61.56)	180 (72.87)		
undergraduate or above	201 (30.54)	154 (37.47)	47 (19.03)		
Married and living together [n (%)]	547 (83.13)	351 (85.40)	196 (79.35)	3.951	0.047
Monthly household income [n (%)]				10.111	0.006
<10,000 yuan	268 (40.73)	148 (36.01)	120 (48.58)		
≥10,000 and <30,000 yuan	364 (55.32)	246 (59.85)	118 (47.77)		
≥30,000 yuan	26 (3.95)	17 (4.14)	9 (3.64)		
Current smoking [n (%)]	77 (11.70)	45 (10.95)	32 (12.96)	0.561	0.454
Current drinking [n (%)]	81 (12.31)	54 (13.14)	27 (10.93)	0.745	0.388
Regular exercise [n (%)]	566 (86.02)	365 (88.81)	201 (81.37)	6.543	0.009
BMI (M ± SD, kg/m^2^)	24.68 ± 3.46	24.79 ± 3.33	24.48 ± 3.67	−1.103	0.271
ADL impaired [n (%)]	41 (6.23)	13 (3.16)	28 (11.34)	16.992	<0.001
Comorbidity index (M ± SD)	2 (1,3)	1 (1,3)	3 (1,4)	7.888	<0.001
SPPB (M ± SD)	9.36 ± 1.58	10.02 ± 1.09	8.26 ± 1.65	−16.365	<0.001
MMSE (M ± SD)	27.66 ± 2.24	28.27 ± 1.52	26.64 ± 2.81	−9.672	<0.001
PHQ-9 (M ± SD)	1 (0,3)	0 (0,2)	2 (0,6)	8.176	<0.001
MNA-SF (M ± SD)	12.97 ± 1.33	13.31 ± 0.92	12.42 ± 1.68	−7.613	<0.001
Vision impaired [n (%)]	161 (24.47)	48 (11.68)	113 (45.75)	95.304	<0.001
Hearing impaired [n (%)]	43 (6.53)	6 (1.46)	37(14.98)	46.456	<0.001
BDNF [M (P_25_, P_75_), µg/L]	3.33 (0.73,9.98)	5.28 (1.59,13.37)	1.22 (0.37,4.21)	−9.371	<0.001
Hs-CRP mg/L, [M (P_25_, P_75_)]	1.47 (0.67,3.29)	1.30 (0.51,3.14)	1.69 (0.87,3.68)	3.076	0.002
IL-6 pg/mL [M (P_25_, P_75_)]	8.03 (3.11,14.93)	8.03 (3.07,14.96)	8.06 (3.19,14.88)	0.375	0.607
TNF-α pg/mL [M (P_25_, P_75_)]	8.89 (3.92,28.21)	9.23 (4.42,37.64)	8.41 (3.08,19.75)	−1.024	0.253
IL-1β pg/mL [M (P_25_, P_75_)]	13.35 (6.89,25.05)	14.77 (7.65,28.51)	11.56 (6.19,20.06)	−3.355	0.001
MDA nmol/mL [M (P_25_, P_75_)]	3.51 (1.04,7.22)	2.91 (0.89,6.56)	4.43 (1.22,8.04)	2.713	0.007
SOD U/mL (M ± SD)	14.36 ± 5.09	14.84 ± 5.74	13.27 ± 4.71	−3.084	0.002
GSH-Px U/mL [M (P_25_, P_75_)]	195.45 (122.18,265.64)	200.65 (118.09, 276.67)	194.72 (127.51,248.52)	−0.691	0.495
GR U/L [M (P_25_, P_75_)]	10.87 (3.49,30.87)	13.39 (4.07,33.21)	5.83 (2.91,22.14)	−3.901	<0.001

Notes: IC, intrinsic capacity; BMI, Body mass index; ADL, Activities of daily living; SPPB, Short physical performance battery; MMSE, Mini mental state examination; PHQ-9, Patient health questionnaire-9; MNA-SF, Mini nutritional assessment-short form; BDNF, Brain-derived neurotrophic factor; Hs-CRP, High sensitivity C-reactive protein; IL-6, Interleukin-6; TNF-alpha, Tumor necrosis factor-alpha; IL-1β, Interleukin-1 beta; MDA, Malondialdehyde; SOD, Superoxide dismutase; GSH-Px, Glutathione peroxidase; GR, Glutathione reductase.

*P*-values were generated using the Student’s *t*-test, Wilcoxon rank sum test, or χ^2^ test.

### 3.2 Associations of BDNF with IC and dimensions

**Fig. 1:**
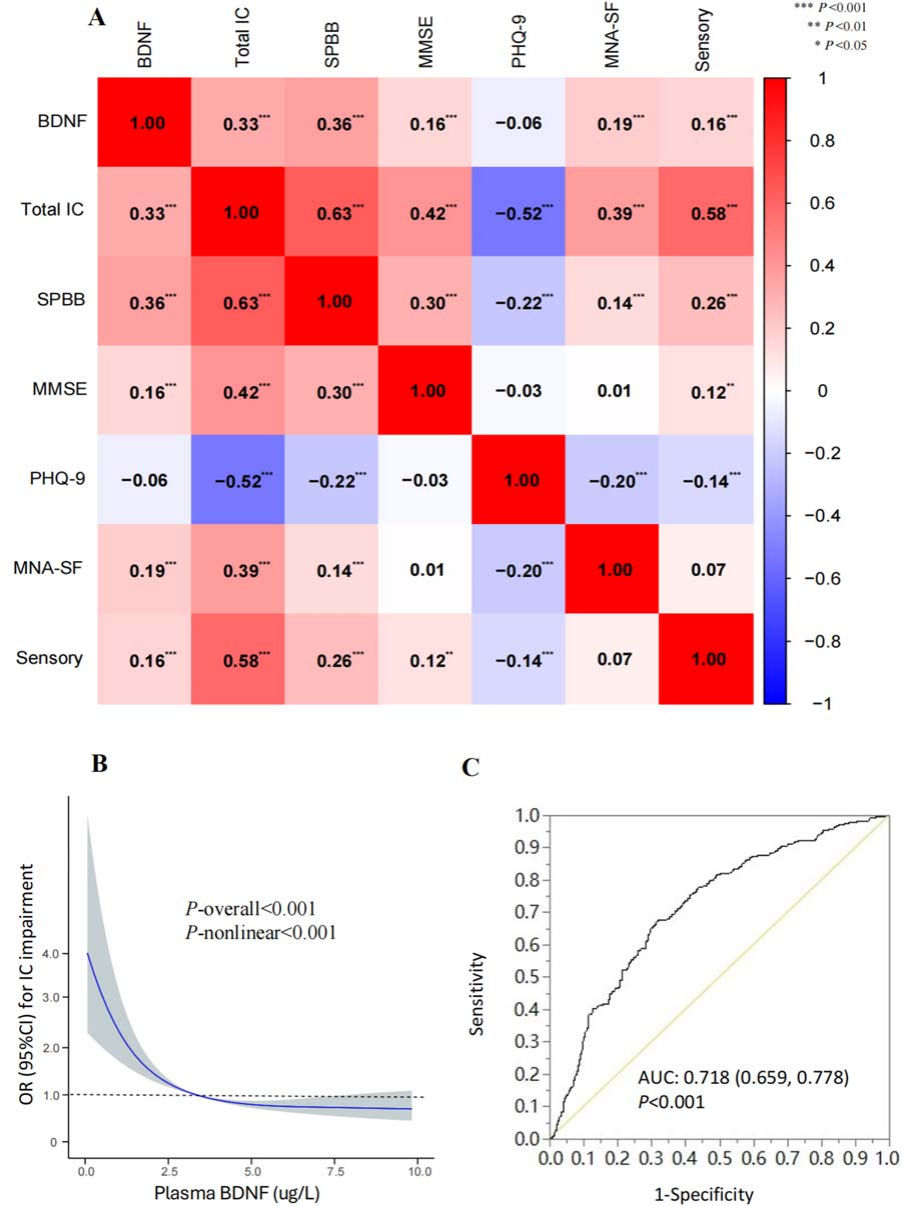
Associations of plasma BDNF with IC (Fig A. Spearman correlations between BDNF levels with IC and dimensions; Fig B. Restricted cubic spline regression analysis of the association between plasma BDNF levels and the risk of IC impairment after adjusting for age, sex, ethnicity, education, household income, marital status, smoking, drinking, regular exercise, BMI, ADL impairment, and comorbidity index; Fig C. ROC curve for BDNF levels in predicting IC impairment.).

Plasma BDNF levels showed a significantly positive moderate correlation with the overall IC score (*r* = 0.33, *P* < 0.001). Specifically, there were significantly positive small to moderate correlations between BDNF and scores of SPPB (*r* = 0.36, *P* < 0.001), MMSE (*r* = 0.16, *P* <0.001), and MNA-SF (*r* = 0.19, *P* <0.001), respectively. No significant correlation was observed between BDNF and PHQ-9 scores (*r* = −0.06, *P* = 0.125). BDNF levels were significantly higher in older individuals with normal vision compared to those with visual impairment [4.28 (0.98, 11.84) µg/L vs. 1.61 (0.36, 6.19) µg/L, Z = −4.845, *P* < 0.001]. No statistically significant difference was observed in BDNF levels between participants with or without hearing impairment (Z = −1.247, *P* = 0.212). The correlations between BDNF levels with IC and each dimension are described in Fig. 1A. Besides, when we adjusted for age, sex, ethnicity, education, household income, marital status, smoking, drinking, regular exercise, BMI, ADL impairment, and comorbidity index, the association between BDNF and the overall IC score remained significant in the multiple linear regression model (*β* = 0.018, SE = 0.005, *P* = 0.003).

### 3.3 Multivariate logistic analyses between BDNF and IC impairment

As shown in Fig. 1B, BDNF levels (as a continuous variable) were negatively associated with the odds of IC impairment with an L-shaped pattern (*P*-overall <0.001, *P*-nonlinear <0.001) in the RCS model. Binary multivariate logistic regression analysis was performed after dividing BDNF into three categorical groups (with the low BDNF group as reference). After adjusting for all confounders, the odds for IC impairment in the medium and high BDNF tertiles were significantly lower than in the low BDNF tertile, with ORs of 0.43(95% CI: 0.26−0.89, *P* = 0.004) and 0.38(95% CI: 0.20−0.71, *P* = 0.007), respectively. The odds of IC impairment decreased with higher levels of BDNF with a *P*-trend of 0.005 (Model 3). The above results were stable in three adjusted models (Table 3). Sex subgroup analyses revealed that the association of BDNF with IC impairment was significant in both men and women with a *P*-interaction of 0.349. As described in Fig. 1C, the AUC for BDNF in predicting IC impairment was 0.718 (95% CI: 0.659−0.778), indicating good predictive ability. The optimal cut-off value of BDNF in the ROC analysis was 2.36 µg/L, with a sensitivity of 0.672 and a specificity of 0.698.

**Table 3:** Multivariate logistic analyses between BDNF levels and IC impairment.

Groups	Participants	IC impairment	Model 1		Model 2		Model 3	
			OR (95% CI)	*P* value	OR (95% CI)	*P* value	OR (95% CI)	*P* value
Overall (n = 658)								
Per 1 unit increase	658	247	0.92 (0.89−0.94)	<0.001	0.93 (0.91−0.95)	<0.001	0.94 (0.91−0.96)	0.001
Low BDNF	219	129	Ref.		Ref.		Ref.	
Medium BDNF	220	79	0.46 (0.29−0.84)	<0.001	0.41 (0.28−0.74)	<0.001	0.43 (0.26−0.89)	0.004
High BDNF	219	39	0.29 (0.15−0.52)	<0.001	0.31 (0.21−0.54)	<0.001	0.38 (0.20−0.71)	0.007
*P*-trend				<0.001		<0.001		0.005
Men (n = 267)								
Per 1 unit increase	267	90	0.94 (0.90−0.97)	0.001	0.94 (0.91−0.98)	0.002	0.94 (0.90−0.98)	0.006
Low BDNF	89	46	Ref.		Ref.		Ref.	
Medium BDNF	89	30	0.55 (0.33−0.91)	0.018	0.47 (0.21−0.88)	0.034	0.55 (0.23−1.13)	0.061
High BDNF	89	14	0.35 (0.22−0.61)	<0.001	0.32 (0.11−0.62)	0.001	0.37 (0.19−0.73)	0.006
*P*-trend				<0.001		0.002		0.003
Women (n = 391)								
Per 1 unit increase	391	157	0.90 (0.86−0.94)	<0.001	0.92 (0.88−0.96)	0.001	0.93 (0.89−0.97)	0.003
Low BDNF	130	84	Ref.		Ref.		Ref.	
Medium BDNF	131	50	0.41 (0.23−0.72)	<0.001	0.38 (0.20−0.67)	<0.001	0.42 (0.16−0.89)	0.011
High BDNF	130	23	0.29 (0.18−0.54)	<0.001	0.27 (0.11−0.56)	0.007	0.34 (0.14−0.85)	0.026
*P*-trend				<0.001		<0.001		0.009

Model 1: adjusting for age and sex (not in sex-subgroup analyses); Model 2: additionally adjusting for ethnicity, education, household income, marital status, smoking, drinking, regular exercise, and BMI; Model 3: additionally adjusting for ADL impairment, and comorbidity index.

IC, intrinsic capacity; BDNF, Brain-derived neurotrophic factor; OR, odds ratio; CI, confidence intervals.

We conducted sensitivity analyses after excluding 41 older individuals with ADL impairment. As shown in Supplement Table S2, we found that plasma BDNF levels exhibited a significant negative association with the odds of IC impairment, with ORs of 0.45 (95% CI: 0.22−0.81, *P* = 0.008) and 0.39 (95% CI: 0.18−0.73, *P* = 0.001) in the medium and high BDNF groups, respectively. In addition, when we included BDNF as a dichotomous variable according to the median value, compared with participants with low BDNF (<3.32 µg/L), those with high BDNF (≥3.32 µg/L) had a 59% (OR: 0.41, 95% CI: 0.22−0.79, *P* = 0.002) reduction in the odds of having IC impairment (Supplementary Table S3). We also used the optimal cut-off value of BDNF (2.36 µg/L) obtained from the ROC analyses as the threshold and found that the odds of BDNF for IC impairment remained stable (Supplementary Table S4).

### 3.4 Mediation analysis

**Fig. 2:**
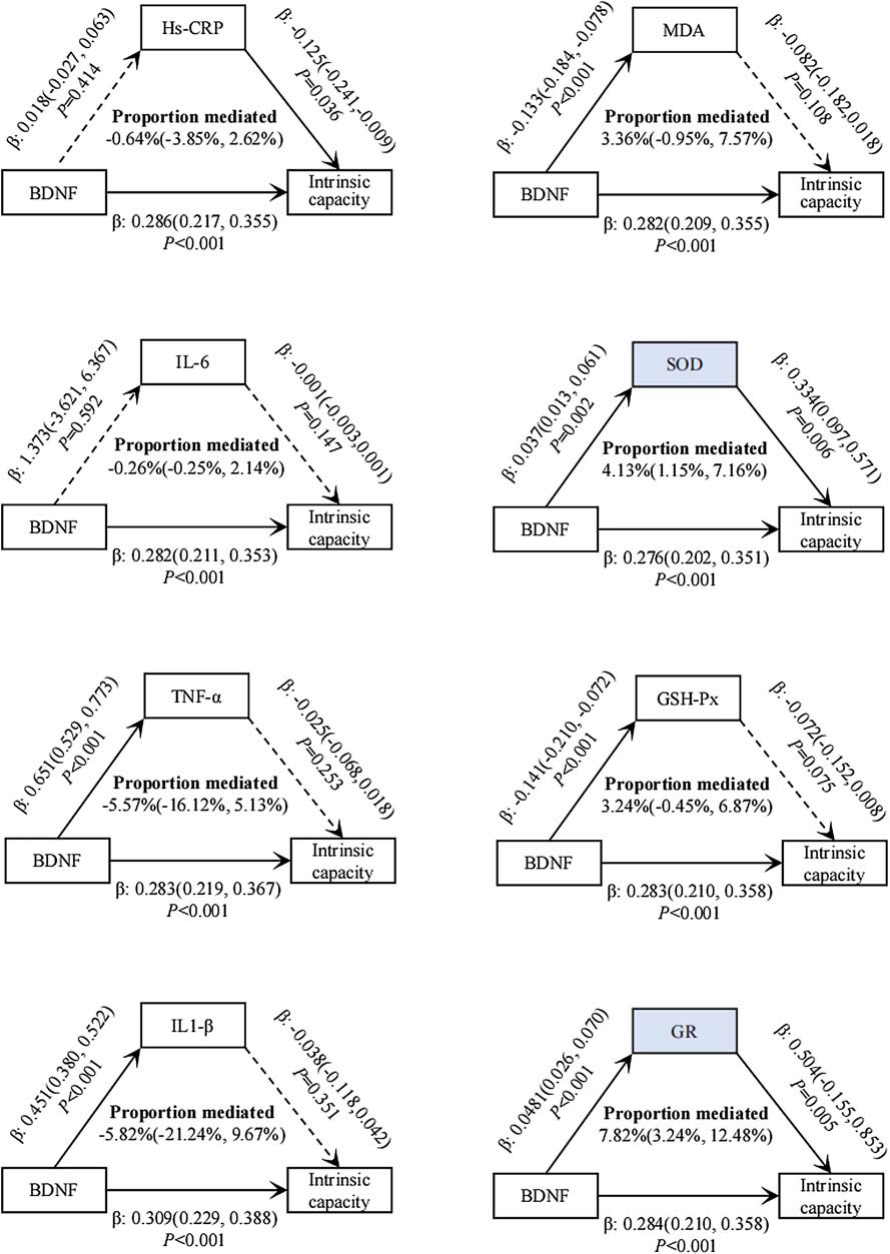
Mediating roles of inflammation and oxidative stress indictors in the relationship between BDNF and IC. Adjusted for age, sex, ethnicity, education, household income, marital status, smoking, drinking, regular exercise, BMI, ADL impairment and comorbidity index. BDNF, Brain-derived neurotrophic factor; Hs-CRP, High sensitivity C-reactive protein; IL-6, Interleukin-6; TNF-alpha, Tumor necrosis factor-alpha; IL-1β, Interleukin-1 beta; MDA, Malondialdehyde; SOD, Superoxide dismutase; GSH-Px, Glutathione peroxidase; GR, Glutathione reductase.

Considering the skewed distribution of the candidate mediators, the inflammatory and oxidative stress markers were log-transformed and included in the models to mitigate the impact of extreme values. As shown in Fig. 2, after adjusting for all confounding factors, the total effect and direct effect of BDNF on IC were 0.289 (95% CI: 0.217−0.361) and 0.275(95% CI: 0.203−0.349), respectively. The indirect effect mediated by SOD was 0.012 (95% CI: 0.004−0.022), and SOD mediated 4.13% (95% CI: 1.15–7.16) of the total effect of BDNF on IC. Similarly, plasma GR mediated 7.82% (95% CI: 3.24–12.48) of the total effect with an indirect effect of 0.024 (95% CI: 0.010−0.039). We did not observe a significant mediating effect of MDA, GSH-Px, and inflammatory markers.

## 4 Discussion

In this study, we employed standardized assessment tools to quantify the dimensions of IC. We found that IC impairment was prevalent among community-dwelling older individuals, with a detection rate of 37.54%. Plasma levels of BDNF were positively correlated with IC, and oxidative stress played a mediating role in this relationship.

Previous studies on the association between BDNF and health outcomes have primarily been based on clinical settings. BDNF has been confirmed to have appropriate predictive value for the risk of frailty and mortality [26,27]. The present study for the first time identified the associations of BDNF with a variety of functional abilities in community-dwelling older individuals, taking into account a series of potential confounders, such as lifestyles and chronic diseases. Specifically, circulating BDNF showed a positive correlation with MMSE scores, which is in line with findings of numerous previous studies. Two foundational studies by Lima [28] and Diniz [29] et al. demonstrated that BDNF activates the PI3K/Akt and MAPK/ERK signaling pathways, enhances neuronal survival, slows down neuronal loss during aging, and may improve learning and memory by boosting synaptic plasticity. A meta-analysis by Li et al. [30] found that peripheral blood BDNF levels were significantly lower in patients with mild cognitive impairment, suggesting that serum BDNF could be a potential biomarker for the early diagnosis of dementia. Kirk and colleagues conducted an MRI analysis of 142 older adults and found a relationship between decreasing levels of BDNF and reduced hippocampal volume of the hippocampus [31]. However, Xie et al. [32] suggested that there was no statistically significant difference in BDNF levels between patients with cognitive impairment and healthy population. The severity of cognitive decline and the presence of comorbidities may influence the role of BDNF. Therefore, the inconsistent findings may be attributed to the heterogeneity of study samples and confounding factors included.

Our research identified a positive correlation between plasma BDNF levels and SPPB scores, indicating that higher BDNF may be associated with enhanced motor function performance in older individuals. Recent research has revealed that skeletal myofibroblasts and endothelial cells can secrete BDNF, which may play specific physiological roles in the skeletal muscle system, including the induction of muscle regeneration, the enhancement of neuromuscular junction stability, and the augmentation of mitochondrial function [13,33].

The existing literature supports the hypothesis that circulating BDNF may be beneficial for the maintenance of the muscle-brain axis, and the related mechanisms have been explored in previous basic research. Chan et al. found that BDNF knockout mice recover endurance more slowly after exercise, and BDNF supplementation can induce metabolic adaptation and enhance mitochondrial function [34]. BDNF can activate the PI3K/Akt/mTOR signaling pathway, promote skeletal muscle protein synthesis, inhibit protein degradation, and thus maintain muscle mass [35]. Nakano et al. found that, in patients with heart failure, BDNF levels were positively correlated with quadriceps strength and 10-meter walking speed, yet showed no significant association with muscle mass [36]. Shi et al. also found that elevated serum BDNF concentrations were positively related to the prevalence of sarcopenia in hemodialysis patients [37]. However, in a recent study, BDNF levels were found to be elevated in community-dwelling older individuals with sarcopenia [19]. The mechanisms of BDNF on muscle loss are complex and need to be further explored. In the present study, higher plasma BDNF levels were associated with better nutritional status. Previous researches have indicated links between BDNF with appetite [38], energy metabolism [34], and a range of psychosomatic disorders [39,40]. These elements are crucial for the assessment of nutrition using MNA-SF. Phillip et al. [41] found that serum BDNF levels in underweight anorexia nervosa patients were significantly lower than in the normal population, and increased gradually with weight regain. In addition, malnourished individuals tend to have insufficient protein intake, which affects the synthesis of precursor proteins of BDNF and thus in turn reduces the level of BDNF.

We also found that BDNF levels in older individuals with visual impairment were significantly lower than those in visually normal individuals. These findings suggested that there might be a negative link between BDNF and aging-related visual loss. In Sato et al’s study, plasma BDNF levels are significantly lower in patients with early to mid-stage glaucoma than in non-glaucomatous patients [42]. BDNF can be synthesized endogenously by retinal ganglion cells, retinal photoreceptor cells and retinal pigment epithelial cells in the retina. Previous experimental studies have demonstrated the involvement of BDNF in the mechanisms of glaucoma, diabetic retinopathy and age-related macular degeneration [23,43]. Although BDNF has been implicated in the development and functional maintenance of the auditory system in vertebrates, our study found no significant association between BDNF levels and auditory dimensions. This may be due to the lack of objective and quantitative hearing detection indexes in the study. The whispered voice test for hearing impairment may have introduced reporting bias. Future research should further explore the effect of BDNF on hearing loss through auxiliary examinations such as pure tone audiometry. BDNF also plays a physiological role in the pathogenesis and treatment of mental disorders through signaling pathways such as BDNF/TrkB, PI3K/PKB, and MAPK [44,45]. However, no association was found between BDNF levels and psychological dimensions in this study. This may be related to the low prevalence of depressive symptoms (3.49%) or mild depressive symptoms (9.73%) in the sample when screened using the PHQ-9 scale. Future studies should increase the sample size to include more cases of depression or systematically analyze the data using different mental illness assessment tools.

Our study explored novel biomarkers associated with IC from the perspective of the brain-muscle axis. Previous researches have confirmed that biomarkers related to the neuromuscular junction, such as the C-terminal agrin fragment and neurofilament light chain, are linked with sarcopenia and neural health [[46], [47], [48]]. Future research should focus on using a panel of circulating markers to provide additional value for IC assessment, beyond that of a single marker. Another advantage of this study is the inclusion of multiple inflammatory and oxidative stress indicators to explore the potential mechanisms of BDNF. We found that in older individuals with impaired IC, MDA levels were higher, while the levels of antioxidant enzymes were lower. Our mediation analysis clarified that SOD and GR mediated 4.14% and 7.82% of the total effect, respectively. Although the mediating role of oxidative stress in the relationship between BDNF and IC was relatively slight, these findings can also help to explain the potential mechanism. Our novel results indicate that the promoting effect of BDNF on IC may be achieved by enhancing cellular antioxidant levels and inhibiting oxidative stress. Elevated reactive oxygen species (ROS) can lead to dysfunction in neuronal and skeletal muscle cells. BDNF exerts neuroprotective effects by inducing the production of antioxidant enzymes that combat oxidative stress and scavenge oxygen free radicals. Through the activation of the PI3K/Akt pathway, BDNF can enhance the synthesis and accumulation of SOD and GSH, thereby boosting the cells' antioxidant capacity [49]. Kwon et al. found that exercise can effectively reduce ROS levels, improve oxidative stress, and upregulate BDNF expression, thus alleviating cognitive impairment and neurodegenerative symptoms caused by oxidative stress [50]. Moreover, although previous studies have confirmed the inhibitory role of BDNF in inflammation [21,51], we did not observe a mediating effect of inflammatory factors. This may be because, compared to hospitalized patients, community-dwelling older individuals did not show a strong inflammatory response. Additionally, we only included hs-CRP, IL-6, TNF-α, and IL-1β. The regulatory effect of BDNF on functional decline may be mediated through other inflammatory markers and related signaling pathways, which requires further exploration.

This study has some limitations. Firstly, the cross-sectional design limits the inferences that can be drawn about the causal relationship between BDNF and IC impairment, suggesting the need for follow-up of IC decline in further longitudinal studies. Secondly, although we have adjusted for demographics, health behaviors, and chronic diseases, we cannot eliminate the potential influence of residual and unmeasured confounding factors. For example, environmental and genetic factors, such as genetic polymorphisms of BDNF, were not considered. Thirdly, our sample was recruited exclusively from communities in Beijing, and the generalizability of the results to other ethnic groups and rural older populations should be approached with caution. Fourth, although we explored the mediating role of oxidative stress between BDNF and IC, it's difficult to clarify the sequence among these biomarkers because the exposure factors and mediating factors were measured at the same time point.

## 5 Conclusion

This study found a positive association between circulating BDNF and IC in community-dwelling older adults, offering novel biological insights into the identification of functional decline. Furthermore, oxidative stress slightly mediates the observed association which may partially elucidate the underlying mechanisms of BDNF.

## CRediT authorship contribution statement

CZ and PZ contributed to the concept and design of the study. CZ, YZ, and YK performed the material preparation and data collection. CZ and JZ performed the data analysis. JL, HS, and SL revised the manuscript. JS provided funding support. All authors have read and agreed to the published version of the manuscript.

## Ethical approval

The study design has been approved by the ethic committee of Beijing Hospital (2022BJYYEC-260-03), and all participants provided signed informed consent prior to the survey.

## Declaration of Generative AI and AI-assisted technologies in the writing process

The authors only used AI to correct the grammar in some sentences.

## Funding

This work was supported by the National High Level Hospital Clinical Research Funding (BJ-2024-219; BJ-2022-133; BJ-2022-149; BJ-2024-157); the National Science and Technology Major Project for the Prevention and Treatment of Cancer, Cardiovascular and Cerebrovascular, Respiratory and Metabolic Diseases (2024ZD0524100; 2024ZD0524101).

## Declaration of competing interest

The authors declare no conflict of interest.

## References

[1] Hoogendijk E.O., Dent E., Koivunen K. (2023). Intrinsic capacity: an under-researched concept in geriatrics. Age Ageing.

[2] Tay L., Tay E.L., Mah S.M., Latib A., Koh C., Ng Y.S. (2023). Association of intrinsic capacity with frailty, physical fitness and adverse health outcomes in community-dwelling older adults. J Frailty Aging.

[3] Stolz E., Mayerl H., Freidl W., Roller-Wirnsberger R., Gill T.M. (2022). Intrinsic capacity predicts negative health outcomes in older adults. J Gerontol A Biol Sci Med Sci.

[4] Campbell C.L., Cadar D., McMunn A., Zaninotto P. (2023). Operationalization of intrinsic capacity in older people and its association with subsequent disability, hospital admission and mortality: results from the english longitudinal study of ageing. J Gerontol A Biol Sci Med Sci.

[5] Sánchez-Sánchez J.L., Lu W.H., Gallardo-Gómez D., Del Pozo Cruz B., de Souto Barreto P., Lucia A. (2024). Association of intrinsic capacity with functional decline and mortality in older adults: a systematic review and meta-analysis of longitudinal studies. Lancet Healthy Longev.

[6] Li X., Ma L. (2024). From biological aging to functional decline: insights into chronic inflammation and intrinsic capacity. Ageing Res Rev.

[7] Giudici K.V., de Souto Barreto P., Guerville F., Beard J., Araujo de Carvalho I., Andrieu S. (2019). Associations of C-reactive protein and homocysteine concentrations with the impairment of intrinsic capacity domains over a 5-year follow-up among community-dwelling older adults at risk of cognitive decline (MAPT Study). Exp Gerontol.

[8] Lu W.H., Guyonnet S., Martinez L.O., Lucas A., Parini A., Vellas B. (2023). Association between aging-related biomarkers and longitudinal trajectories of intrinsic capacity in older adults. Geroscience.

[9] Lu W.H., Gonzalez-Bautista E., Guyonnet S., Lucas A., Parini A., Walston J.D. (2023). Plasma inflammation-related biomarkers are associated with intrinsic capacity in community-dwelling older adults. J Cachexia Sarcopenia Muscle.

[10] Doi T., Makizako H., Tsutsumimoto K., Hotta R., Nakakubo S., Makino K. (2018). et al. Association between Insulin-Like Growth Factor-1 and Frailty among Older Adults. J Nutr Health Aging.

[11] Lana A., Valdés-Bécares A., Buño A., Rodríguez-Artalejo F., Lopez-Garcia E. (2017). Serum leptin concentration is associated with incident frailty in older adults. Aging Dis.

[12] Li X., Cao X., Ying Z., Yang G., Hoogendijk E.O., Liu Z. (2022). Plasma superoxide dismutase activity in relation to disability in activities of daily living and objective physical functioning among Chinese older adults. Maturitas.

[13] Delezie J., Weihrauch M., Maier G., Tejero R., Ham D.J., Gill J.F. (2019). BDNF is a mediator of glycolytic fiber-type specification in mouse skeletal muscle. Proc Natl Acad Sci.

[14] Notaras M., van den Buuse M. (2019). Brain-Derived Neurotrophic Factor (BDNF): novel insights into regulation and genetic variation. Neuroscientist.

[15] Oudbier S.J., Goh J., Looijaard S., Reijnierse E.M., Meskers C.G.M., Maier A.B. (2022). Pathophysiological mechanisms explaining the association between low skeletal muscle mass and cognitive function. J Gerontol A Biol Sci Med Sci.

[16] Copeland E.N., LeBlanc P.J., Duarte-Guterman P., Fajardo V.A., MacPherson R.E.K. (2025). The link between sarcopenic obesity and Alzheimer’s disease: a brain-derived neurotrophic factor point of view. J Physiol.

[17] Collins J.M., Hill E., Bindoff A., King A.E., Alty J., Summers M.J. (2021). Association between components of cognitive reserve and serum BDNF in healthy older adults. Front Aging Neurosci.

[18] Mizoguchi Y., Yao H., Imamura Y., Hashimoto M., Monji A. (2020). Lower brain-derived neurotrophic factor levels are associated with age-related memory impairment in community-dwelling older adults: the Sefuri study. Sci Rep.

[19] Pratt J., Motanova E., Narici M.V., Boreham C., De Vito G. (2025). Plasma brain-derived neurotrophic factor concentrations are elevated in community-dwelling adults with sarcopenia. Age Ageing.

[20] Miyazaki S., Iino N., Koda R., Narita I., Kaneko Y. (2021). Brain-derived neurotrophic factor is associated with sarcopenia and frailty in Japanese hemodialysis patients. Geriatr Gerontol Int.

[21] Noren Hooten N., Ejiogu N., Zonderman A.B., Evans M.K. (2015). Protective effects of BDNF against C-reactive protein-induced inflammation in women. Mediators Inflamm.

[22] Ma L., Zhang Y., Liu P., Li S., Li Y., Ji T. (2021). Plasma N-terminal Pro-B-Type natriuretic peptide is associated with intrinsic capacity decline in an older population. J Nutr Health Aging.

[23] Ghaffariyeh A., Honarpisheh N., Heidari M.H., Puyan S., Abasov F. (2011). Brain-derived neurotrophic factor as a biomarker in primary open-angle glaucoma. Optom Vis Sci.

[24] Binford S.S., Hubbard E.M., Flowers E., Miller B.L., Leutwyler H. (2018). Serum BDNF is positively associated with negative symptoms in older adults with schizophrenia. Biol Res Nurs.

[25] Piancatelli D., Aureli A., Sebastiani P., Colanardi A., Del Beato T., Del Cane L. (2022). Gene-and gender-related decrease in serum BDNF levels in Alzheimer’s disease. Int J Mol Sci.

[26] Ritter C., Miranda A.S., Giombelli V.R., Tomasi C.D., Comim C.M., Teixeira A.L. (2012). Brain-derived neurotrophic factor plasma levels are associated with mortality in critically ill patients even in the absence of brain injury. Crit Care.

[27] Roh E., Hwang S.Y., Song E., Park M.J., Yoo H.J., Baik S.H. (2022). Association of plasma brain-derived neurotrophic factor levels and frailty in community-dwelling older adults. Sci Rep.

[28] Lima Giacobbo B., Doorduin J., Klein H.C., Dierckx R.A., Bromberg E., de Vries E.F. (2019). Brain-derived neurotrophic factor in brain disorders: focus on neuroinflammation. Mol Neurobiol.

[29] Diniz B.S., Teixeira A.L. (2011). Brain-derived neurotrophic factor and Alzheimer’s disease: physiopathology and beyond. Neuromol Med.

[30] Li Z., Guo J., Zhang X., Zhong A., Fang X., Cheng Z. (2023). Meta-analysis of the association between peripheral blood brain-derived neurotrophic factor and Alzheimer’s disease. Chin J Behav Med Brain Sci.

[31] Erickson K.I., Prakash R.S., Voss M.W., Chaddock L., Heo S., McLaren M. (2010). Brain-derived neurotrophic factor is associated with age-related decline in hippocampal volume. J Neurosci.

[32] Xie B., Zhou H., Liu W., Yu W., Liu Z., Jiang L. (2020). Evaluation of the diagnostic value of peripheral BDNF levels for Alzheimer’s disease and mild cognitive impairment: results of a meta-analysis. Int J Neurosci.

[33] Zhang Z., Wang B., Fei A. (2019). BDNF contributes to the skeletal muscle anti-atrophic effect of exercise training through AMPK-PGC1α signaling in heart failure mice. Arch Med Sci.

[34] Chan W.S., Ng C.F., Pang B.P.S., Hang M., Tse M.C.L., Iu E.C.Y. (2024). Exercise-induced BDNF promotes PPARδ-dependent reprogramming of lipid metabolism in skeletal muscle during exercise recovery. Sci Signal.

[35] Rodríguez-Gutiérrez E., Torres-Costoso A., Saz-Lara A., Bizzozero-Peroni B., Guzmán-Pavón M.J., Sánchez-López M. (2024). Effectiveness of high-intensity interval training on peripheral brain-derived neurotrophic factor in adults: a systematic review and network meta-analysis. Scand J Med Sci Sports.

[36] Nakano I., Kinugawa S., Hori H., Fukushima A., Yokota T., Takada S. (2020). Serum brain-derived neurotrophic factor levels are associated with skeletal muscle function but not with muscle mass in patients with heart failure. Int Heart J.

[37] Shi Q., Fan F. (2024). Diagnostic value of triglyceride-glucose index and brain—derived neurotrophic factor for sarcopenia in non-diabetic maintenance hemodialysis patients. Chin J Blood Purif.

[38] Stanek K., Gunstad J., Leahey T., Glickman E., Alexander T., Spitznagel M. (2008). Serum brain-derived neurotrophic factor is associated with reduced appetite in healthy older adults. J Nutr Health Aging.

[39] Zou Y., Zhang Y., Tu M., Ye Y., Li M., Ran R. (2024). Brain-derived neurotrophic factor levels across psychiatric disorders: a systemic review and network meta-analysis. Prog Neuropsychopharmacol Biol Psychiatry.

[40] Hao L.S., Du Y., Chen L., Jiao Y.G., Cheng Y. (2022). Brain-derived neurotrophic factor as a biomarker for obsessive-compulsive disorder: a meta-analysis. J Psychiatr Res.

[41] Phillips K.E., Jimerson D.C., Pillai A., Wolfe B.E. (2016). Plasma BDNF levels following weight recovery in anorexia nervosa. Physiol Behav.

[42] Phillips K.E., Jimerson D.C., Pillai A., Wolfe B.E. (2023). Reduced plasma BDNF levels in normal tension glaucoma compared to open angle glaucoma. J Glaucoma.

[43] Phillips K.E., Jimerson D.C., Pillai A., Wolfe B.E. (2021). Differential expression of BDNF and BIM in streptozotocin-induced diabetic rat retina after fluoxetine injection. In vivo.

[44] Gao L., Zhang Y., Sterling K., Song W. (2022). Brain-derived neurotrophic factor in Alzheimer’s disease and its pharmaceutical potential. Transl Neurodegener.

[45] Wang C.S., Kavalali E.T., Monteggia L.M. (2022). BDNF signaling in context: from synaptic regulation to psychiatric disorders. Cell.

[46] Pratt J., Motanova E., Pessanha L., Narici M., Boreham C., De Vito G. (2024). Plasma C-terminal agrin fragment concentrations across adulthood: reference values and associations with skeletal muscle health. J Cachexia Sarcopenia Muscle.

[47] Giacomucci C., Mazzeo S., Bagnoli S., Ingannato A., Leccese D., Berti V. (2022). Plasma neurofilament light chain as a biomarker of Alzheimer’s disease in Subjective Cognitive Decline and Mild Cognitive Impairment. J Neurol.

[48] Pratt J., De Vito G., Segurado R., Pessanha L., Dolan J., Narici M. (2022). Plasma neurofilament light levels associate with muscle mass and strength in middle-aged and older adults: findings from GenoFit. J Cachexia Sarcopenia Muscle.

[49] Kim D.H., Li H., Yoo K.Y., Lee B.H., Hwang I.K., Won M.H. (2007). Effects of fluoxetine on ischemic cells and expressions in BDNF and some antioxidants in the gerbil hippocampal CA1 region induced by transient ischemia. Exp Neurol.

[50] Kwon D.H., Kim B.S., Chang H., Kim Y.I., Jo S.A., Leem Y.H. (2013). Exercise ameliorates cognition impairment due to restraint stress-induced oxidative insult and reduced BDNF level. Biochem Biophys Res Commun.

[51] Lima Giacobbo B., Doorduin J., Klein H.C., Dierckx R., Bromberg E., de Vries E.F.J. (2019). Brain-derived neurotrophic factor in brain disorders: focus on neuroinflammation. Mol Neurobiol.

