# Supplementary material for: Cross-sectional association between brain-derived neurotrophic factor and intrinsic capacity in older adults: The mediating role of oxidative stress
Source: J Nutr Health Aging. 2025 Jun 12;29(8):100599. doi: 10.1016/j.jnha.2025.100599 (PMC12402360; doi:10.1016/j.jnha.2025.100599)
Supplement: Supplementary file 1 [file mmc1.docx]

Effects and Persistence of *Bifidobacterium animalis* subsp. *lactis* BL-99 and Fructooligosaccharides on Older Adults with Functional Constipation: A Randomized, Double-Blind, Placebo-Controlled Trial

**Supplementary materials**

**I. Supplementary methods**

1. Inclusion and Exclusion Criteria

This study focused on a population of generally healthy older individuals who experienced no other significant comorbidities apart from functional constipation.

**Inclusion Criteria (Must meet all the following criteria):**

(1) Age ≥ 60 years;

(2) Body mass index 18.5–34.9 kg/m2[1];

(3) Diagnosed with functional constipation according to the Rome IV criteria [2]:

(i) Presence of at least two of the following symptoms:

(a) Straining during more than 25% of defecation;

(b) Lumpy or hard stools (Bristol Stool Form Scale types 1–2) for more than 25% of defecation;

(c) Sensation of incomplete evacuation for more than 25% of defecation;

(d) Sensation of anorectal obstruction/blockage for more than 25% of defecation;

(e) Manual maneuvers to facilitate more than 25% of defecation (e.g., digital evacuation, pelvic floor support);

(f) Fewer than three spontaneous bowel movements per week.

(ii) Loose stools are rarely present without the use of laxatives;

(iii) Fulfillment of these criteria for the past three months and onset of symptoms over six months prior to the diagnosis.

(4) Willingness to participate in the study and provision of a signed informed consent form

**Exclusion Criteria (participants were excluded if they met any of the following criteria):**

(1)Diagnosis of organic diseases, such as colorectal cancer, colorectal or anal stenosis, rectal mass or fecal impaction, pseudo-obstruction, megarectum, or other serious gastrointestinal diseases (e.g., stomach or duodenal ulcers, acute enteritis, ulcerative colitis);

(2)History of major gastrointestinal surgery (e.g., gastrectomy, gastrointestinal anastomosis, intestinal resection);

(3)Severe cerebrovascular diseases, neurological disorders, endocrine/metabolic diseases, or infectious diseases[3];

(4)Use of the following medications within the last month: prokinetic agents, H2 receptor antagonists, proton pump inhibitors, antispasmodics, opioid-containing drugs, chronic non-steroidal anti-inflammatory drugs[4], or antibiotics;

(5)Regular consumption (≥3 times per week) of probiotic or prebiotic products, such as yogurt or probiotic powder;

(6)Significant abnormalities in blood pressure (having a systolic blood pressure of ≥160mmHg or a diastolic blood pressure of ≥110mmHg)[5] or blood tests, or severe anemia;

(7)Excessive smoking (≥20 cigarettes per day)[6] or frequent heavy alcohol consumption (≥40 grams of alcohol per day);

(8)Participation in other clinical studies within the last month;

(9)Any other condition deemed inappropriate for participation by the researchers.

**Withdrawal Criteria:**

(1) Allergic reactions to the test substances;

(2) Poor compliance, lack of cooperation with follow-up or treatment, or voluntary withdrawal;

(3) Participants met the inclusion criteria but failed to take the test substances as prescribed, affecting the assessment of efficacy;

(4) Serious complications or adverse reactions during the study.

2. 16S Amplicon Sequencing

Amplicon sequencing was conducted on a PacBio platform. Genomic DNA was extracted and used for PCR amplification of the full-length 16S rRNA gene. The amplified products from multiple samples were pooled in equal amounts, then subjected to damage repair, end repair, adapter ligation, enzyme digestion, and fragment selection using BluePippin. A dumbbell-shaped library was obtained and underwent quality control prior to sequencing on the PacBio platform. The resulting sequence data were analyzed and interpreted. The amplicons were sequenced and analyzed by BGI (Shenzhen, China).

1. **Gut microbiota analysis**

The data obtained from sequencing were processed for quality control, and low-quality sequences were removed. An RDP classifier (16S rRNA data species classification tool) was used to annotate each sequence with the species classification. For the gut microbiota data analysis, α diversity indices, including Sobs, ACE, Shannon, and Simpson, were calculated based on operational taxonomic units. The differences between the groups were analyzed by applying a non-parametric analysis of variance (ANOVA) with the Mann-Whitney U Test. The β diversity was determined by PCoA based on the Unweighted_unifrac distance algorithm, and permutational multivariate analysis of variance (PERMANOVA, also named Adonis analysis) was used to analyze the significant difference between groups. Linear discriminant analysis (LDA) of effect size (LEfSe) was determined for the bacterial taxa with different abundances in the placebo and synbiotics groups before and after the intervention. The significance level was set at LDA ≥ 2.0 and *P*<0.05 for the LEfSe analyses. Spearman’s correlation was conducted to analyze the correlation between phenotypic indexes of constipation and gut microbiota.

**II. Supplementary results**

**Table S1. Baseline characteristics of participants**

|  | **Placebo**  **（n = 33）** | **Synbiotics**  **（n = 34）** | ***P*** |
| --- | --- | --- | --- |
| Gender（men，%） | 12（36.3） | 12（35.3） | 0.927 |
| Age（years） | 68.24 ± 3.12 | 68.24 ± 3.09 | 0.993 |
| BMI（kg/m^2^） | 24.28 ± 2.25 | 24.23 ± 3.01 | 0.708 |
| Frequency of spontaneous bowel movements per week | 3.09±1.28 | 3.14±1.20 | 0.854 |
| Drug use to improve constipation（n，%） | 7（21.2） | 8（23.5） | 0.820 |
| Whole gut transit time（hour）* | 50.90±18.40 | 55.50±24.50 | 0.942 |
| Rome IV criteria |  |  |  |
| Criterion 1: Sensation of incomplete evacuation in more than 1/4 defecation（n，%） | 24（72.7） | 30（88.2） | 0.231 |
| Criterion 2: Sensation of anorectal obstruction/blockade in more than 1/4 defecation（n，%） | 21（63.6） | 20（58.8） | 0.686 |
| Criterion 3: Manual maneuvers to facilitate more than 1/4 defecation（n，%） | 4（12.1） | 6（17.6） | 0.526 |
| Criterion 4: Straining during a bowel movement in more than 1/4 defecation（n，%） | 11（33.3） | 12（35.3） | 0.866 |
| Criterion 5: Hard or lumpy stools in more than 1/4 defecation（n，%） | 20（60.6） | 23（67.6） | 0.548 |

Data are presented as mean ± standard deviation or n (%). * The number of participants who completed the baseline intestinal transit time assessment in the placebo and synbiotic groups was 20 and 25, respectively. The placebo group was given maltodextrin, synbiotic group was given a synbiotic composed of BL-99 (2×10^10^ CFU/day) and fructooligosaccharides. Intergroup differences were analyzed using independent sample t-tests or chi-square tests. BMI, body mass index.


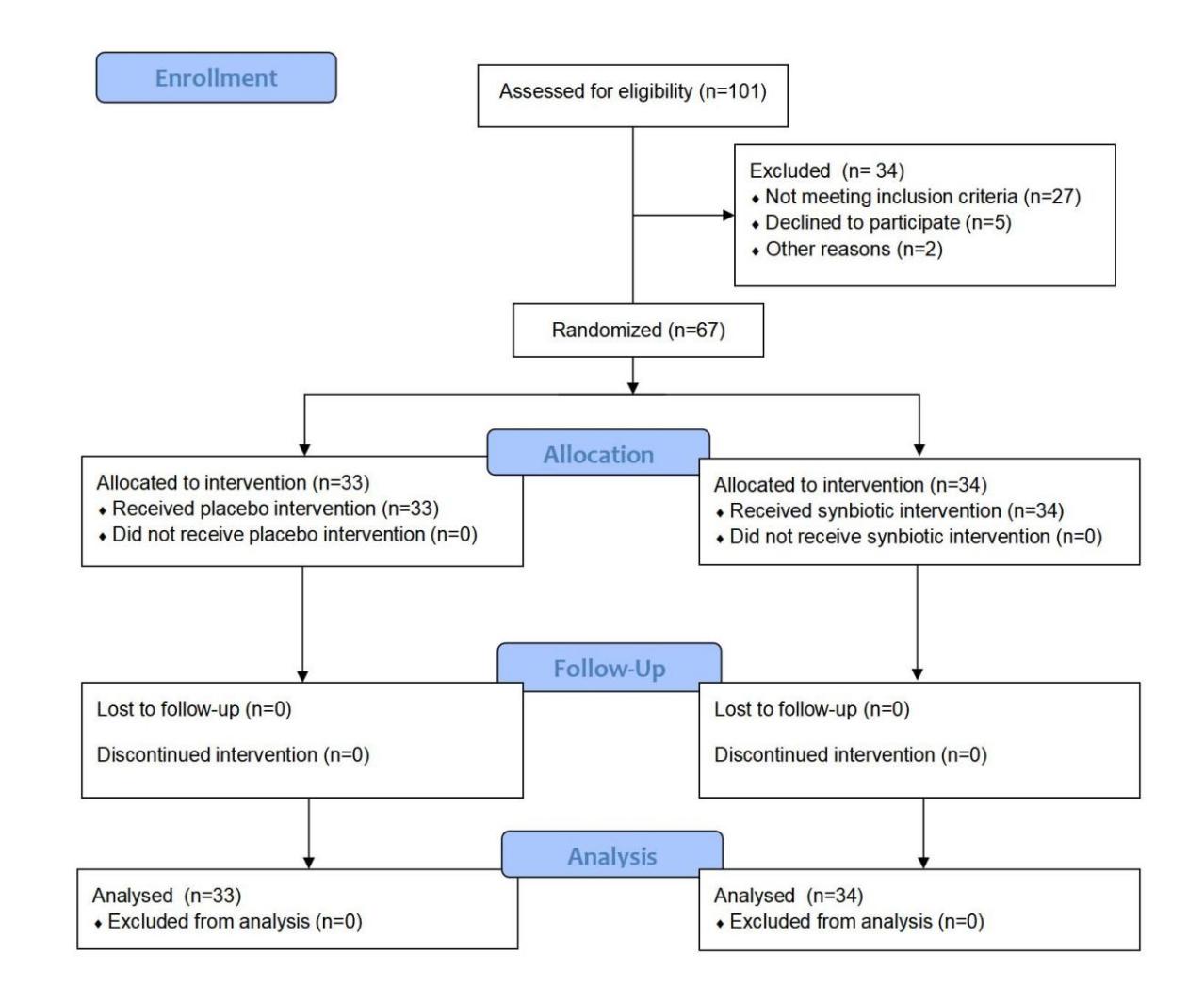


**Figure S1 Flow diagram**

**Table S2. Effect of synbiotic intervention on stool consistency**

| **Stool Type** | **V0** | | **V1** | | **V2** | |
| --- | --- | --- | --- | --- | --- | --- |
|  | **Placebo**  **(n=33)** | **Synbiotics**  **(n=34)** | **Placebo**  **(n=33)** | **Synbiotics**  **(n=34)** | **Placebo**  **(n=33)** | **Synbiotics**  **(n=34)** |
| **Hard**  **(Types 1–2)** | 22(66.7%) | 21(61.8%) | 11(33.3%) | 11(32.4%) | 9(27.3%) | 14(41.2%) |
| **Normal**  **(Types 3–5)** | 8(24.2%) | 9(26.5%) | 19(57.6%) | 19(55.9%) | 20(60.6%) | 16(47.1%) |
| **Loose**  **(Types 6–7)** | 3(9.1%) | 4(11.8%) | 3(9.1%) | 4(11.8%) | 4(12.1%) | 4(11.8%) |
| ***P*** | 0.900 | | 0.938 | | 0.468 | |

Data are presented as n (%). Intergroup differences were analyzed using chi-square tests. V0, V1 and V2 represent the baseline, the end of the intervention, and the follow-up period ended, respectively. Hard (Types 1-2) was defined as constipation, Normal (Types 3-5) as normal defecation, and Loose (Types 6-7) as diarrhea.

**Table S3. Least squares means and standard errors of symptom scores assessed by PAC-SYM questionnaire between the placebo and synbiotic groups**

| Symptoms | V0 （0 week） | | *P* | V1 (intervention for 4 weeks) | | *P* | V2 (2 weeks after intervention ended) | | *P* |
| --- | --- | --- | --- | --- | --- | --- | --- | --- | --- |
|  | Placebo | Synbiotics |  | Placebo | Synbiotics |  | Placebo | Synbiotics |  |
|  | (n=33) | (n=34) |  | (n=33) | (n=34) |  | (n=33) | (n=34) |  |
| Bowel movement that were too hard | 2.15±0.16 | 1.71±0.16 | 0.056 | 2.33±0.16 | 1.88±0.16 | 0.054 | 2.30±0.16 | 1.94±0.16 | 0.120 |
| Bowel movement that were too small | 1.76±0.16 | 1.82±0.16 | 0.766 | 2.15±0.16 | 2.15±0.16 | 0.984 | 1.94±0.16 | 1.85±0.16 | 0.696 |
| Reduction in bowel movements | 1.64±0.19 | 1.53±0.19 | 0.695 | 1.48±0.19 | 1.74±0.19 | 0.359 | 1.24±0.19 | 1.44±0.19 | 0.466 |
| Straining or squeezing to try to pass bowel movements | 1.88±0.18 | 1.29±0.18 | 0.025 | 2.18±0.18 | 2.44±0.18 | 0.316 | 1.97±0.18 | 1.88±0.18 | 0.735 |
| Painful bowel movements | 1.85±0.19 | 2.09±0.19 | 0.378 | 0.97±0.19 | 1.15±0.19 | 0.514 | 1.12±0.19 | 0.74±0.19 | 0.157 |
| Incomplete bowel movement like you did not finish | 0.73±0.18 | 0.97±0.18 | 0.338 | 1.88±0.18 | 2.09±0.18 | 0.409 | 1.73±0.18 | 1.53±0.18 | 0.435 |
| Feeling like you had to pass a bowel movement but you could not | 1.58±0.20 | 2.00±0.20 | 0.131 | 1.91±0.20 | 1.82±0.20 | 0.760 | 1.70±0.20 | 1.12±0.20 | 0.040 |
| Rectal bleeding or tearing during or after bowel movement | 1.33±0.16 | 1.74±0.15 | 0.071 | 0.42±0.16 | 0.35±0.15 | 0.747 | 0.61±0.16 | 0.12±0.15 | 0.029 |
| Rectal burning during or after a bowel movement | 0.15±0.15 | 0.29±0.15 | 0.506 | 0.67±0.15 | 0.65±0.15 | 0.927 | 0.79±0.15 | 0.21±0.15 | 0.007 |
| Pain in your abdomen | 0.24±0.13 | 0.21±0.13 | 0.843 | 0.45±0.13 | 0.29±0.13 | 0.387 | 0.45±0.13 | 0.44±0.13 | 0.942 |
| Bloating in your abdomen | 0.36±0.14 | 0.41±0.14 | 0.811 | 0.58±0.14 | 0.68±0.14 | 0.617 | 0.61±0.14 | 0.44±0.14 | 0.413 |
| Stomach cramps | 0.45±0.17 | 0.35±0.17 | 0.673 | 1.42±0.17 | 1.35±0.17 | 0.767 | 1.09±0.17 | 0.85±0.17 | 0.324 |

Data are the least squares means±standard errors of each group calculated using the linear mixed effects model. The *P* values represent the differences between groups at each visit. PAC-SYM, Patient assessment of constipation-symptoms.

**Table S4. Least squares means and standard errors of symptom scores assessed by CSS questionnaire between the placebo and synbiotic groups**

| Symptoms | V0 （0 week） | | *P* | V1 (intervention for 4 weeks) | | *P* | V2 (2 weeks after intervention ended) | | *P* |
| --- | --- | --- | --- | --- | --- | --- | --- | --- | --- |
|  | Placebo | Synbiotics |  | Placebo | Synbiotics |  | Placebo | Synbiotics |  |
|  | (n=33) | (n=34) |  | (n=33) | (n=34) |  | (n=33) | (n=34) |  |
| Frequency of bowel movements | 0.94±0.11 | 0.62±0.10 | 0.032 | 0.39±0.11 | 0.44±0.10 | 0.75 | 0.36±0.11 | 0.35±0.1 | 0.943 |
| Difficulty: painful evacuation effort | 2.48±0.16 | 2.71±0.16 | 0.326 | 1.91±0.16 | 2.15±0.16 | 0.29 | 1.61±0.16 | 1.94±0.16 | 0.137 |
| Completeness: feeling incomplete evacuation | 3.12±0.16 | 3.38±0.15 | 0.235 | 2.88±0.16 | 3.29±0.15 | 0.060 | 2.73±0.16 | 2.62±0.15 | 0.618 |
| Pain: abdominal pain | 2.27±0.15 | 2.47±0.15 | 0.353 | 2.18±0.15 | 2.03±0.15 | 0.474 | 2.24±0.15 | 2.24±0.15 | 0.973 |
| Time: minutes in lavatory per attempt | 2.70±0.15 | 2.79±0.15 | 0.643 | 2.61±0.15 | 2.12±0.15 | 0.021 | 2.61±0.15 | 2.09±0.15 | 0.014 |
| Assistance: type of assistance | 0.33±0.11 | 0.53±0.11 | 0.206 | 0.18±0.11 | 0.29±0.11 | 0.468 | 0.09±0.11 | 0.29±0.11 | 0.190 |
| Failure of evacuation: unsuccessful attempts for evacuation per 24 hours | 0.82±0.13 | 1.06±0.12 | 0.173 | 0.85±0.13 | 0.65±0.12 | 0.254 | 0.79±0.13 | 0.53±0.12 | 0.144 |

Data are the least squares means±standard errors of each group calculated using the linear mixed effects model. The *P* values represent the differences between groups at each visit. CSS, Constipation Scoring System.

**
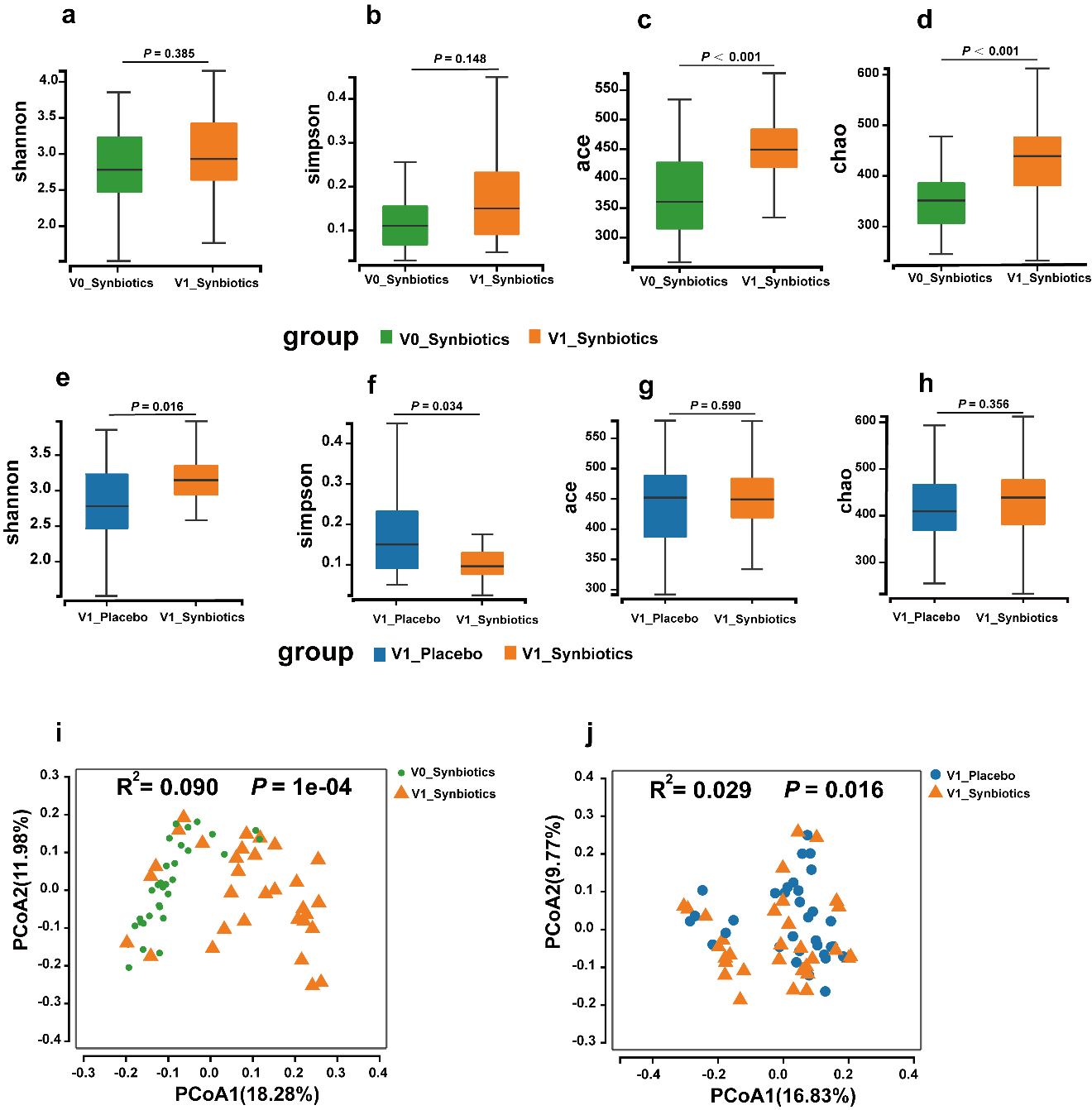
**

**Figure S2. Effect of BL-99 and FOS synbiotic intervention on gut microbiota diversity in older adults with functional constipation**

α-diversity indexes including shannon (a, e), simpson (b, f), ace (c, g),and chao (d, h). Principal coordinate analysis (PCoA) based on the OTU levels using unweighted_unifrac distances (i, j)

**References**

[1] Sun X, Zhang S, Zhou X. A causal association between obesity and constipation: a two-sample bidirectional Mendelian randomization study and meta-analysis. Frontiers in nutrition 2024;11:1430280,10.3389/fnut.2024.1430280.

[2] Lee JH, Kim GB, Han K, Jung EJ, Suh HJ, Jo K. Efficacy and safety of galacto-oligosaccharide in the treatment of functional constipation: randomized clinical trial. Food Funct 2024;15(12):6374-6382,10.1039/d4fo00999a.

[3] Chen J, Gao X, Liang J, Wu Q, Shen L, Zheng Y, et al. Association between gut microbiota dysbiosis and poor functional outcomes in acute ischemic stroke patients with COVID-19 infection. mSystems 2024;9(6):e0018524,10.1128/msystems.00185-24.

[4] Beppu K, Osada T, Shibuya T, Watanabe S. [Pathogenic mechanism of NSAIDs-induced mucosal injury in lower gastrointestinal tract]. Nihon rinsho. Japanese journal of clinical medicine 2011;69(6):1083-1087

[5] Ishiyama Y, Hoshide S, Mizuno H, Kario K. Constipation-induced pressor effects as triggers for cardiovascular events. Journal of clinical hypertension (Greenwich, Conn.) 2019;21(3):421-425,10.1111/jch.13489.

[6] Coulie B, Camilleri M, Bharucha AE, Sandborn WJ, Burton D. Colonic motility in chronic ulcerative proctosigmoiditis and the effects of nicotine on colonic motility in patients and healthy subjects. Alimentary pharmacology & therapeutics 2001;15(5):653-663,10.1046/j.1365-2036.2001.00959.x.
